# Lateral violence and lateral empowerment in relation to young Indigenous populations across the CANZUS nations: a scoping review

**DOI:** 10.1080/00049530.2025.2521787

**Published:** 2025-07-06

**Authors:** Yvonne Clark, Feda Ali, Anneka Bowman, Rachel Elovaris, Sarah Clark, Treena Clark, Karen L. Peterson, Naomi Thornthwaite, Imani Austin, Karen Hawke

**Affiliations:** a Aboriginal Communities and Families Health Research Alliance (ACRA) SAHMRI, Adelaide, South Australia, Australia; b Discipline of Psychology, within Justice and Society, University of South Australia, Adelaide, South Australia, Australia; c School of Psychology, University of Adelaide, Adelaide, South Australia, Australia; d Stillbirth Centre for Research Excellence, Mater Research, University of Queensland, South Brisbane, Queensland, Australia; e Molly Wardaguga Institute for First Nations Birth Rights, Charles Darwin University, Darwin, Northern Territory, Australia; f Faculty of Design and Architecture, University of Technology, Sydney, New South Wales, Australia; g Department of Public Health, Flinders University, Adelaide, South Australia, Australia

**Keywords:** Lateral empowerment, lateral violence, youth, Indigenous, child, CANZUS

## Abstract

**Objective:**

Young Indigenous people are vulnerable to lateral violence, where negative feelings and behaviours are directed towards members within their own oppressed group. Lateral empowerment, in contrast, is where young people can promote resilience and strength and collectively prevent and repair the effects of lateral violence. The aim of this scoping review was to describe how young Indigenous people aged between 0 and 25 years across the CANZUS nations (Canada, Australia, New Zealand, and the United States) experience lateral violence particularly relating to the context and forms in which young people are experiencing it; and what mechanisms of lateral empowerment pertaining to healing programmes and interventions, address young Indigenous people’s healthy wellbeing?

**Methods:**

A scoping review was conducted using the Joanna Briggs Institute (JBI) framework to search for peer-reviewed publications between January 1980 and May 2024. In total, 170 peer-reviewed publications were thematically mapped into nine themes to provide an overview of the existing literature.

**Results:**

Four themes to describe how young people experience lateral violence were identified: Familial and/or community violence; Interpersonal violence victimization (IVV); Peer and gang violence and Self-harm behaviour. Five themes related to the mechanisms of lateral empowerment which included: Community informed treatment approaches; Cultural identity and resilience; Family wellbeing; Use of digital technology; and Trauma intervention.

**Conclusion:**

Young Indigenous people are experiencing lateral violence in many forms. Fortunately, empowering strategies were identified that strengthen young people’s wellbeing, cultural identity, connectedness to community and country to mitigate the negative impacts of lateral violence were identified.

## Key points

**What is already known about this topic:**

1. Lateral violence is a phenomenon that occurs across the world to all people, particularly those prone to oppression such as minority groups, Indigenous peoples and within professions such as nursing.
2. Many Indigenous peoples have been subject to intergenerational and transgenerational trauma via invasion and colonization, leaving an oppressive legacy with a negative discourse.
3. Lateral violence has been reported within Indigenous communities, with young people often the most vulnerable.

**What the topic adds:**

1. A thorough map of the information pertaining to lateral violence and lateral empowerment for young Indigenous people across the CANZUS nations.
2. Greater awareness of approaches to lateral empowerment available to young Indigenous people.

## Background

Lateral (sideways, within-group or horizontal) violence has been described as the way people experiencing powerlessness, direct their rage, fear, shame, anger, and dissatisfaction inwards towards themselves and outwardly towards others within their own systemically exploited and oppressed group (Clark et al., 2016). This means that frustrations, negative behaviours and feelings can be directed towards the self in the form of self-harm and/or vented upon peers, family, and one’s community rather than confronting the systems that oppresses them (Bombay et al., 2014; Jaber et al., 2023; Whyman et al., 2022). The conceptualization of lateral violence (LV) originally emerged from key discourse on the impacts of colonization and slavery on affected populations in countries such as Africa and South America (Fanon, 1967; Freire, 1970). Tuck and Yang discuss the different forms of colonization in which settlers seek to “destroy and disappear the Indigenous people that live there” (pg. 6), by claiming and exporting Indigenous resources (external colonialism) and controlling Indigenous people via prisons, schooling and government policies (internal colonialism) (Tuck & Yang, 2012). In the Australian context, a history of colonization and dispossession of Aboriginal and Torres Strait Islander peoples has created a legacy of oppression and racism that continues to negatively impact individuals, communities, and whole populations. Whereby, the oppressed who feel powerless, can become the oppressors (Dudgeon et al., 2000) and be suspended in a state of fear as suggested by Freire (1970, p. 47): “the oppressed having internalised the image of the oppressor and adopted his guidelines are fearful of freedom”.

Racism as a form of oppression has a considerable influence on Social and Emotional Wellbeing (SEWB) outcomes for young people, and in which their cultural identity is positioned within a colonial society. Chelsea Watego demonstrates her lived negative experiences of racism and oppression in an Australian context in her renowned book (Watego, 2021). A longitudinal Australian study on Aboriginal children aged 5–10 years demonstrated an increased risk of asthma and obesity for children experiencing direct and prolonged racism (C. C. Shepherd et al., 2017). Young people are particularly vulnerable to LV, racism, and trauma, especially in relation to health and wellbeing outcomes (Bailey, 2020; Charles & DeGagné, 2013; Coffin, 2011; Herrenkohl et al., 2022). Marcia Langton has argued that for Aboriginal people in Australia “Those most at risk of lateral violence in its raw physical form [overt violence] are family members and, in the main, the most vulnerable members of the family: old people, women and children. Especially the children” (Langton, 2008, p. 50). A systematic review by Priest et al. (2013) found a strong and consistent positive relationship between racial discrimination and negative mental health outcomes for young people, such as anxiety, depression, and psychological distress. Witnessing and experiencing racism and oppression can be internalized and normalized at a very young age, leaving children and adolescents at risk emotionally, mentally, spiritually, and physically. Cumulative and generational effects of racism along with LV can have dire consequences for young people with one of the most serious consequences being self-harm and suicide within Indigenous communities (Please note that, herein, the word “Indigenous” is used to refer to collectively First Nations people from any colonized country and “Aboriginal” will refer to Aboriginal and Torres Strait Islander people in Australia) (Chan et al., 2023). Findings consistently show high rates of suicide among Indigenous young people across the CANZUS nations. For example, in Australia, the percentage of deaths due to suicide between 2018 and 2022 was highest among Aboriginal young people aged 0–24 years, suicide accounted for nearly one-quarter (22.0%) of all recorded deaths (AIHW). First nations American Indian and Māori males and females aged 15–24 years report suicide at double the rates than their non-Indigenous counterparts (Coroners Court: Te Koti Kaitirotiro Matewhawhati, 2024; Xu et al., 2021). Between 2011 and 2016 the suicide rates for Indigenous Canadian youth aged between 15 and 24 years were also significantly higher in comparison to non-Indigenous youth (Suicide among First Nations people, Métis and Inuit, 2011–2016).

Although young people are the most vulnerable to LV, they are also the future leaders and change makers; in an Australian context “the future of Aboriginal communities lies with the next generation and therefore a focus on prevention and unity needs to start with young Aboriginal people” (Clark et al., 2017a, p. 112). At present, there is limited guidance on how to manage, address, and rectify LV within Aboriginal and Torres Strait Islander communities, especially when LV is relational to other phenomena such as racism, discrimination, trauma and wellbeing. Some documented coping mechanisms to deal with LV and the associated trauma include avoidant behaviours, such as not identifying with one’s cultural background (Bennett, 2014) and disengaging from family, community, school, and Aboriginal workplaces (Clark et al., 2017a). In contrast, empowering coping strategies can include support from family, workplace and counselling, positive role modelling and directly challenging LV (Clark et al., 2017b) and arming oneself with knowledge and awareness of LV through education (Clark & Glover, 2020). Strengthening one’s connection to culture, community and identity, changing attitudes towards LV by understanding the underlying mechanisms driving the behaviour and encouraging systematic changes that promote the self-determination and unity of people not division of power can also increase coping capacity for Indigenous people (Whyman et al., 2024).

Empowerment, thriving and flourishing are intertwined with young people’s wellbeing and balance. In Australia, SEWB, is a term used to describe a holistic approach to building and nurturing collective and cultural identities (Dudgeon et al., 2023; Durmush et al., 2021; Graham Gee et al., 2014). Adult and child focused SEWB models recognize that strengths (i.e., protective factors) and challenges (i.e., risk factors) to domains are interconnected and occur within the contexts of historical, social, economic, and political determinants (Gee et al., 2014; Priest et al., 2012). For adults, seven SEWB domains have been described as spirituality/ancestry, body/behaviours, mind/emotions, family/kinship, country/land, community, and culture (TIMHW, 2021). In Canada, Indigenous people utilize a medicine wheel for their wellbeing that represents self, family, community, nation, society, and ecology of creation to understand the nature of balance, harmony, and mino-bimaadiziwin – “our way of life” (mzinegiizhigo-Kwe Bédard, 2018). LV has been shown to negatively impact Aboriginal people’s spiritual wellbeing by weakening one’s spirit reducing their ability to connect with their spiritual wellbeing (Whyman et al., 2021). Disconnection to these wellbeing domains or when there is an imbalance and disruption to harmony renders individuals and communities vulnerable. Vulnerability can manifest physically, such as poor physical health, drug abuse, and eating disorders, and emotionally, such as behavioural difficulties, which increases the likelihood of ongoing mental health issues for children into adulthood (Walker et al., 2014) and potentially increases LV within communities.

In Australia, lateral empowerment (LE) has emerged as a term to describe efforts to foster autonomy and self-determination. This is about enabling young Indigenous people to have control in their lives and support their peers to do the same, particularly to make their own decisions and choices that counteract or decrease the prevalence of LV in their context (Newton, 2016; Priday et al., 2011). LE contributes to and appears to be synonymous with holistic and circular concepts of wellbeing and harmony among Indigenous peoples (Gee et al., 2014). Therefore, strengthening and nurturing such connections within these wellbeing and harmony frameworks, at an individual, collective, or systems level, within family, community, and work contexts, facilitates cultural and emotional safety in various environments and can enable a sense of flourishing within Indigenous communities (Dudgeon et al., 2023).

### No gold standard universal definition of lateral violence and lateral empowerment

A widely used international definition of LV is described as “the way powerless people covertly and overtly direct their dissatisfaction inward, towards each other, towards themselves and towards those less powerful than themselves” (Native Counselling Services of Alberta [NCSA], as cited in Clark & Augoustinos, 2015, p. 27). The definition is broad and inclusive of any type of violence to the self (including self-harm) or within one’s own oppressed group and is used in diverse settings relating to any oppressed groups.

LV can be experienced within diverse settings transcending oppressed groups impacted by colonization, and LV, as defined here, has been used in studies for at least the past four decades to examine dysfunctional interactions (e.g., workplace incivilities) among nurses, who are considered to be an oppressed group within their workplace due to a power imbalance within the medical profession hierarchy (Roberts, 2015). LV, while present in various settings, manifests differently in Indigenous communities than within the nursing profession. This is due to the nature and context of LV within Indigenous communities being embedded within the historical, societal and political context of oppression and associated behaviours. Within the nursing profession, LV often involves conflict and control of power between staff and peers and situated within a work context. Within Indigenous communities, LV is underpinned by colonialism, historical trauma and cultural identity and often intertwined within intergenerational family and community relationships which are a daily lived experience for which Indigenous people are unable to disconnect from with the resulting cumulative compounding nature of the impacts of LV causing immense psychological harm.

LV as a broad term and concept includes a large variety of behaviours and phenomena within various contexts that are often discussed in isolation to LV. For example, in an Indigenous context, research on overt, individual or subgroup behaviours and phenomena such as suicide, self-harm, and interpersonal, domestic, family, community, and gang violence are rarely linked conceptually to LV (Jaber et al., 2023). Whereas more covert forms such as gossiping, shaming, bullying, family feuding, jealousy (Coffin, 2011), and attempting to denigrate another’s cultural identity (Clark et al., 2016), are more likely to be linked with the term LV in the literature (Clark & Augoustinos, 2015).

The concept of “within group” fighting and oppression whereby hampering the success of others due to one’s insecurities has been around for a lengthy period with related terms such as “tall poppy” (Peeters, 2003), “crabs in the bucket” (Chapman, 2022) and “Divide and Conquer” (Posner et al., 2009) dating back to ancient Roman times. The term “lateral violence” has been used in Australia and globally to refer to adults for almost three decades and is rarely used in the context of research related to Indigenous children and young people (Clark et al., 2016; Jaber et al., 2023; Whyman et al., 2021).

**Figure 1.**
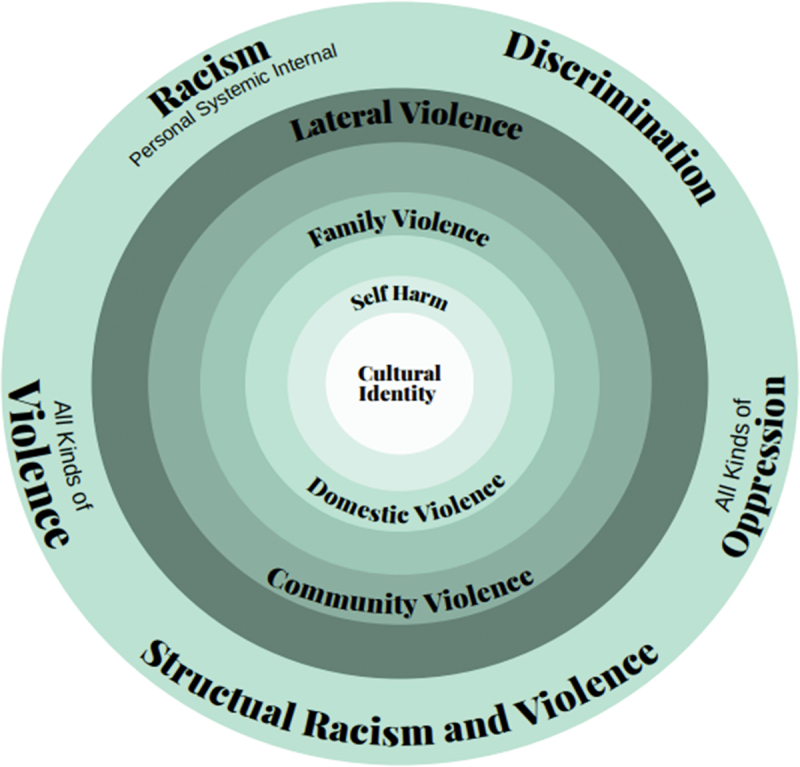
Layers of lateral violence (2024).

Another way to describe LV is via layers or contexts. Figure 1 is a diagram often used in consultation with Aboriginal groups in South Australia (Clark, 2024), it shows cultural identity as a core component of the self and the circles around it as the different layers that LV can be perpetrated within Indigenous contexts. It is explained that LV can be perpetrated on the self, in the home, and in the family. LV is a broader concept and can be perpetrated in any of the layers as well as the local or broader community. The outer ring shows the related and oppressive practices that can trigger LV within these contexts. As LV can be perpetrated at multiple levels, the impact of LV is also layered. Challenges to one’s sense of cultural identity (Aboriginal identity) at the core of LV can lead to people questioning their sense of self and their belonging in community, leading to disconnection from community creating fractures to the learning and transmission of cultural knowledge, cultural practices, language and cultural values making them vulnerable to being lost (Whyman et al., 2021).

There is no universal definition of lateral empowerment (LE), and it is seen as a sense of autonomy or self-determination. This includes being able to have control over one’s life, having agency over choices by an individual or a collective to transform into positive actions and outcomes (Alsop & Heinsohn, 2005). Broadly, we define LE as the way Indigenous peoples can protect, support, and care for each other to reverse or eliminate LV, and promote resilience and strength. We postulate that LE is the process and outcome by which Indigenous communities, families, and young people, empower each other to eliminate the cycle of LV, and promote resilience, healing and strength (Newton, 2016; Priday et al., 2011).

### Purpose of the scoping review

A comprehensive scoping method was used to map the peer-reviewed literature and answer the following research questions: Firstly, how are young Indigenous peoples in the CANZUS nations (Canada, Australia, New Zealand, and the United States) experiencing LV particularly relating to the context and forms in which young people are experiencing it; and secondly, what mechanisms of LE pertaining to healing programmes and interventions, address young Indigenous people’s healthy wellbeing?

There is a paucity of adult or young person’s research which focuses on the experience of LV and that of which use the term LV or how to empower people and mitigate the negative psychological and SEWB impacts. Thus far, a scoping review by Whyman et al. (2022) focused upon the experience of LV within the adult population that examined the psychosocial impacts and identified the need for further research relating to health and wellbeing, and a systematic review by Jaber et al. (2023) focused on the lived experience of Indigenous women experiencing LV, through the lens of intersectionality and post-colonial theories. Our scoping review differs in various ways in that it specifically focuses on young Indigenous populations in the CANZUS nations; it includes elements relating to empowerment and healing strategies for young Indigenous people and it uses broader terminology rather than only focusing on the term “lateral violence” which is a relatively new term and therefore may not identify incidences of oppressed group infighting and within group violence that maybe labelled as another form or layer of violence (as per Figure 1).

The CANZUS nations have been chosen because Indigenous people in these countries have similar lived experiences of colonization, racism, discrimination and inter and transgenerational trauma (Priday et al., 2011). The trauma experienced by Indigenous peoples in CANZUS nations is deeply embedded in historical and contemporary oppressive colonialism (Blackstock et al., 2020; Jaber et al., 2023). The authors acknowledge that the Indigenous groups within each of the CANZUS nations have been categorized as homogenous groups within this scoping review and that the within-group similarities and differences in experiences of LV and LE have not been examined.

For this review, a comprehensive scoping method was used with broad search terms within many contexts and forms and behaviours (i.e., overt and covert) to capture alternative and related experiences and incidences that both LV and LE have been reported and termed in the literature (Colquhoun et al., 2014). The scoping review seeks to explore and conceptualize in-group violence and what this looks like for young Indigenous people in the literature. In the adult population in Australia, alternative terms and conceptualizations for LV have also been used within Aboriginal and Torres Strait Islander communities. For example, LV has been known by many colloquial or local terms (i.e., Black Politics, Black Poppy syndrome, internalized racism); hence, it would be impossible to identify all the relevant LV literature using only the term “lateral violence” alone (Clark & Augoustinos, 2015). Relational terms, behaviours and outcomes related to both LV and LE were included in a robust data search.

## Methods

### Protocol

The objectives, inclusion criteria and methods for this scoping review, were specified in advance and documented in a protocol drafted using the Joanna Briggs Institute (JBI) Manual for Evidence Synthesis available from https://synthesismanual.jbi.global, congruent with the Preferred Reporting Items for Systematic Reviews and Meta-analysis Protocols (PRISMA). The JBI methodology for scoping reviews combined with the Arksey and O’Malley five-step framework was used to guide our research: (1) identification of research question/concept, (2) identification of relevant articles, (3) study selection, (4) charting data, and (5) collating, summarizing, and reporting results (Arksey & O’Malley, 2005). The scoping review purpose, aim and research question are aligned with the Population, Concept and Context (PCC) framework as per the JBI method of review (M. D. Peters et al., 2015). In accordance with a scoping review methodology, we have reported the main findings of the studies but did not infer, interpret synthesis, or make conclusions about these findings rather used the findings to identify and thematically map the available evidence. This is contrasted to a systematic review which reports statistics and effect sizes to answer a clinically meaningful question or to provide evidence to inform practice (Munn et al., 2018).

### Eligibility criteria

Studies were included if there was a focus on Indigenous children, adolescents, and young adults aged between 0 and 25 years from the CANZUS nations (Canada, Australia, New Zealand, and the United States). The search terms encompassed a broad list of terminology used across the various nations within the CANZUS countries. These search terms are related to concepts and terminology used to describe LV and LE in the literature (see Table 1). Peer-reviewed quantitative and qualitative articles, programme evaluations, and reviews reporting direct, related or vicarious experiences of LV or LE within Indigenous communities were included if they were published between January 1980 and May 2024 inclusive. Settings eligible for inclusion were across the four CANZUS nations, including but not limited to, justice systems, government organizations, workplaces, schools, community settings, families, homes, and health services. Studies were excluded if 1) they were not reported in the English language, 2) the Indigenous population was less than 5% of the study population, 3) they were not focused on young people or families where young people were impacted, or 4) full text was unable to be obtained.

**Table 1.** Search strategy.

(1) Outline of search terms and search strategy	
**Population**: Indigenous, Aboriginal and/or Torres Strait Islander **Ages**: 0–25 years **Geographic locations**: Canada, Australia, New Zealand, US (CANZUS) **Search terms for both Lateral Violence and Lateral Empowerment**: Oceanic Ancestry Group, Indigenous people*, Aborigin*, Indigenous*, Māori*, Metis*, Eskimo*, Inuit*, American native continental ancestry group, Samoan*, Chamoros*, Cook Islander, Tongan*, Niuean*, Fijian*, Tokelauan*, Tuvaluan*, Native American*, American Indian*, Canadian Indian*, Torres Strait Island*, first nation*, first people*, Native Hawaiian*, Native Alaskan*, Alaska native*, arctic people*, first American*, first Australian, first Canadian. **AND** young adult, child, infant, young people*, teen*, youth, adolescent*, babies, baby, newborn, toddler*, Rangatahi*, children*. **THEN** lateral violence, horizontal violence, horizontal oppression, internal oppression, lateral oppression, internal* racism, intra* racism, intra-racial racism, ingroup racism, intra* bullying, intra-racial bullying, domestic violence*, famil* violence*, famil* abuse*, intra* violence*, intra-racial violence, intra-cultural violence, intra* abuse, intergenerational violence, intergenerational abuse, infighting, cultural violence, cultural abuse, community violence, spiritual violence, spiritual abuse, internalized colonialism, internalized colonialism, cultural identity. **OR** lateral love*, lateral empowerment*, lateral healing*, lateral respect*, lateral forgiveness*, lateral positivity*, lateral kindness*, lateral wellbeing*, lateral understanding*, lateral strength*, intra* respect*, intra* love*, intra* healing*, commun* healing*, commun* love*, commun* empowerment*, commun* forgiveness*, commun* respect*. **Language**: English **Years of publication**: 1 January 1980 to 31 May 2024	
(2) Number of hits per database searched	
Database	# of hits (citations) – LV and/or LE
CINAHL	299
IBSS	483
Medline	263
ProQuest	476
PsycInfo	308
PubMed	225
Sage	600
Scopus	498
Web of Science	332
Total found: Duplicates removed: **Final citation list for screening:**	3221 1087 **2134**

### Search strategy

**Figure 2.**
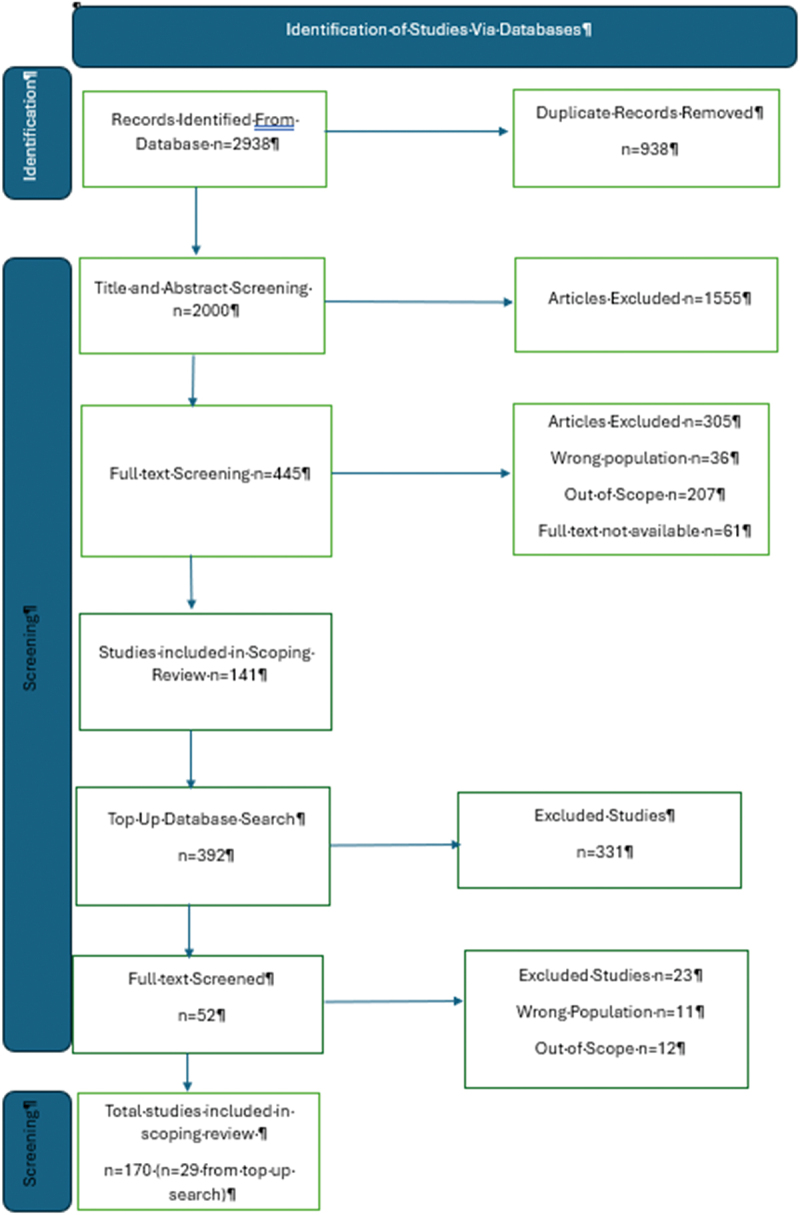
PRISMA-P search and appraisal flowchart. PRISMA-P = Preferred reporting items for systematic reviews and meta-analysis protocols. Adapted from Jaber et al., (2023).

To identify relevant articles for the study, the following bibliographic databases were searched: MEDLINE, PsycINFO, CINAHL, PubMed, IBSS, Web of Science, ProQuest, Sage, and Scopus. The search strategies were drafted by KH and YC and can be found in Table 1. The reference lists of all identified articles were searched for additional studies. All identified peer-reviewed citations were collated and uploaded into Covidence, an online systematic review application (Veritas Health Innovation, Melbourne, Australia), and duplicates were removed. Initially, 141 studies were included in the scoping review, and a further top-up search was conducted in May 2024 which yielded a further 29 studies. In total, 170 studies were used in this scoping review. Figure 2 shows the database search and selection process.

### Selection and screening of sources of evidence

Seven reviewers formed the screening team (KH, RE, FA, YC, AB, SC, NT) and reviewed the title and abstract of 282 publications in Covidence independently. The titles and abstracts were independently screened by two reviewers against the inclusion/exclusion criteria. Pairs of reviewers resolved conflicts by discussion, and where conflict could not be resolved, a third senior reviewer was consulted for resolution. Potentially relevant sources were retrieved in full and assessed against the inclusion criteria by two independent reviewers, and the conflict resolution process above was employed for any conflicts in screening full texts. Reasons for exclusion are identified in Figure 2.

### Data charting process

A data extraction form was designed by the team of reviewers (Appendix I). Extracted information included but was not limited to: 1) synonyms for LV and LE, 2) concepts/triggers that underpin LV/LE (e.g., LV: oppression, racism, intergenerational trauma, LE: identity, resilience), 3) behaviours that identify LV/LE (e.g., LV: bullying, covert/overt violence, LE: good mental health, help seeking behaviour), 4) experiences and impacts of LV/LE (e.g., LV and LE: mental and physical wellbeing, healing) and 5) impact on health and SEWB outcomes, particularly family relations, child and adolescent health and wellbeing development and functioning, criminal behaviours, spiritual health and wellbeing, and connection to culture and identity.

All seven reviewers (KH, RE, FA, YC, AB, SC, NT) extracted data which was then assessed for consensus by two Indigenous reviewers, using their cultural knowledge, lived experience and scholarly expertize in LV and LE. The authors acknowledge the availability of the formalized CREATE quality appraisal tool for assessing research and articles in Australian settings with Aboriginal and Torres Strait Islander people developed by Hartfield et al. (2020). This tool is primarily used to assess the quality of studies and articles for systematic reviews which aims to answer research questions based upon a rigid set of factors. Scoping reviews adopt a broader approach to map out the literature which is not based upon a rigid set of factors; hence, a formal assessment of the methodological quality of studies is not performed in scoping reviews (M. D. Peters et al., 2015). However, some quality appraisal factors that correspond with the CREATE tool were incorporated in the data extraction form which collated information pertaining to participants’ indigeneity and in the lateral empowerment article searches focused on using strength-based studies which demonstrated capacity to empower Aboriginal and Torres Strait Islander participants. As most eligible articles were qualitative, the extracted data were synthesized thematically using Microsoft Excel. The first author (YC) is an Indigenous Scholar with expertize in LV and provided guidance of the analysis and writing of this scoping review. We reported four main themes around LV and five themes around LE. Theme tables can be found in Appendix II, which summarize the setting, population, participant numbers, and study design. The number of studies under each theme ranged from 4 to 78, and common themes and meanings arising from the literature were grouped between LV and LE where possible. As a scoping review, we report the main findings of the studies, but we do not infer, interpret, synthesize, or make conclusions about these findings.

## Results

In total, 170 peer-reviewed articles were included in this scoping review, and the findings of the grey literature have been published elsewhere (Clark et al., 2024). The articles in this review were dated between 1997 and 2024; of those, 33 articles were quantitative studies, 105 articles were qualitative, and 32 articles were mixed methodology. The articles have been further broken down into Lateral Violence and Lateral Empowerment for each CANZUS nation as per below in Table 2.

**Table 2.** Descriptives of articles in scoping review.

	LV	LE	Total
Australia	15	30	*N*=45
Canada	6	24	*N*=30
New Zealand	13	11	*N*=24
United States	24	43	*N*=67
CANZUS	0	4	*N*=4
**Total**	**58**	**112**	**N=170**

### Lateral violence as experienced by young Indigenous people

The review found few results that used the term “lateral violence” directly but rather described forms of ingroup fighting and violence such as: family violence, peer victimization, dating violence, peer and gang violence, community violence and self-harm behaviour. Four main themes emerged from these works to reflect young Indigenous people’s experience of LV which included: familial and/or community violence, interpersonal violence victimization, peer and gang violence, and self-harm behaviours.

### Familial and/or community violence

A total of 30 studies across the CANZUS nations explored familial and/or community violence as experienced by young Indigenous people (Appendix II, Table A1). Many of these studies explored the impact of violence exposure on Indigenous young people within their home and community. Exposure to violence from family and community was found to be associated with poor emotional and behavioural outcomes and violent offending (Bearinger et al., 2005; Fanslow et al., 2021; Frost, 2014; Ioane & Lambie, 2016; Ioane et al., 2014; Lim et al., 2012; Marie et al., 2009; McKinney, 2003; McNulty & Bellair, 2003; Noble-Carr et al., 2019; Sarche et al., 2009); diagnosis of mental health disorders (Basu & Isaacs, 2019; Dickerson & Johnson, 2012; Hautala & Sittner, 2021; Kaspar, 2013; Nalls et al., 2009; Willmon-Haque & Bigfoot, 2008); suicidal and self-harming behaviours (Matthay et al., 2019); and substance misuse (Coleman et al., 2001; Hautala & Sittner, 2021; Short et al., 2019). Poor health outcomes and physical injuries were identified to occur for Indigenous children exposed to family and/or community violence (Askew et al., 2013; Linton & Kim, 2014; Orr et al., 2020). Impaired parenting capacity and fractured parental relationships were found to be associated with violence exposure in the home (Cripps et al., 2009; Meyer & Stambe, 2022; Probst et al., 2008; Ryan & Wilson, 2010; Wilson et al., 2004). The studies also revealed that young LGBTQ+ people were particularly vulnerable to family violence (Soldatic et al., 2023).

### Interpersonal violence victimization (IVV)

Fourteen studies explored experiences of interpersonal violence victimization (IVV) through peer relationships for Indigenous youth (Appendix II, Table A2). Characteristics of the IVV identified included physical and sexual violence, dating violence, and bullying. Young Indigenous members of the lesbian, gay, bisexual, transgender, queer or questioning (LGBTQ+) community were vulnerable to IVV, particularly sexual and dating violence (Du Mont et al., 2017; Edwards et al., 2021; Fix et al., 2021). Young Indigenous people who experienced IVV required hospital admissions for injuries and poor mental health, with girls and young women most at risk of death (Berry et al., 2009; Dhunna et al., 2021; Meuleners et al., 2010; Oetzel & Duran, 2004; Short et al., 2019). Feelings of jealousy, misunderstandings about relationship status, and alcohol consumption among Aboriginal and Torres Strait Islander young people were drivers for IVV towards young women within their community (Senior et al., 2017; Shore & Spicer, 2004). Young people experiencing IVV were shown to be at greater risk of perpetrating violent behaviours towards others, including sexual offending and school violence (Andreescu & Overstreet, 2020; Grey et al., 2023; Meuleners et al., 2009, 2010; Rodney et al., 2005).

### Peer and gang violence

Nine studies examined the influence of peer and gang relationships on young Indigenous people’s behaviours (Appendix II, Table A3). Several of these studies explored peer gang affiliation for young Indigenous people in the United States (Bell & Lim, 2005; Gillies et al., 2017; McNulty & Bellair, 2003; Peacock & Albert, 2000; Scott & Dearing, 2012). It was speculated that gang affiliation was used by young people to replace fractured family systems (Peacock & Albert, 2000) and to create social and emotional support to deal with the pains of poverty, racism, and social isolation (Bell & Lim, 2005). Gang affiliation was also found to be associated with engagement in violent behaviours at school (Donnermeyer et al., 2000; Gazis et al., 2010). Peer relationships were found to be important among Indigenous young people and when fractured contributed to lateral violence. For example, the mental health of Aboriginal and Torres Strait Islander and Māori students was found to be negatively impacted by peer rejection (Kaspar, 2013). Indigenous Canadian university students experiencing LV by their Indigenous peers were found to disengage from university studies and not associate with other Indigenous students to avoid LV (Bailey, 2020). One study in the review found that Aboriginal and Torres Strait Islander youth who identified more strongly with their cultural identity were more susceptible to LV and peer problems (McAlister et al., 2024).

### Self-harm behaviours

The review identified seven studies relating to self-harm, in particular suicidal behaviour among young Indigenous people (Appendix II, Table A4). While LV could be viewed as a risk factor for suicide, it was also seen as a form of LV and a manifestation of young people’s experiences of LV. Suicide completion in 2010 was found to be 1.5 to 4 times higher for Indigenous youth across the CANZUS nations, compared to non-Indigenous youth and the leading cause of hospitalization and death for Alaskan Native youth (Aho & Liu, 2010). Suicide attempts were in 2012 the leading cause of hospitalization and death for Alaskan Native youth (Wexler et al., 2012). A systematic review exploring literature from across the CANZUS nations reported a correlation between experiencing abuse during childhood and suicide later in life (Hatcher et al., 2017), while an American study showed higher rates of suicide among American Indian high-school students who experienced IVV as either a victim or a perpetrator (Else et al., 2009). A disrupted sense of identity and belonging was a risk factor for suicide among Inuit boys and young men in Arctic Canada (Kral, 2012), as was residing in rural and remote areas (Freedenthal & Stiffman, 2004; Hill et al., 2020).

### Forms of lateral empowerment

The literature demonstrated that Indigenous young people across the CANZUS nations have an incredible capacity for healing, learning, and adapting. The results described a diversity of efforts within Indigenous communities and families to empower young people to address LV along with other issues in a holistic manner and promote resilience and strength for young people. We categorized descriptions of LE into five main themes.

### Community informed treatment approaches

Thirteen studies emphasized the importance of community-informed treatment approaches to minimize the impacts of LV on Social and Emotional Wellbeing (SEWB) among Indigenous youth (Appendix II, Table A5). This includes decolonization of programmes and adaptation or co-creation of programmes through strong tribal and academic partnerships (O’Keefe et al., 2019; Trout et al., 2018; Wexler et al., 2012). These studies identified the importance of a holistic approach for programmes aimed at preventing and addressing family violence; these involved young people and their families and encompassed components of family and community strengthening, cultural teachings, peer mentoring, spiritual and emotional experiences (DeRiviere, 2018; Taylor et al., 2004). Teaching effective parenting strategies promoted safe and nurturing relationships and home environments for those exposed to violence in the home (Gee et al., 2020; McWhirter, 2010). Strong family and peer relationships enabled Indigenous youth to become more resilient in the face of social stressors, decreased the risk of post-traumatic stress disorder symptoms among youth exposed to violence, and reduced the risk of family violence (Kral et al., 2014; Taonui, 2010; Zahradnik et al., 2010). Community-based interventions embedded within cultural knowledge and practices are effective when addressing substance use and poor mental health in Indigenous youth (Dickerson & Johnson, 2011; Komro et al., 2023; Rothman et al., 2022).

### Cultural identity and resilience

Eighty studies demonstrated the importance of strengthening cultural identity and resiliency as a protective factor for Indigenous youth and children (Appendix II, Table A6). Community-informed interventions targeting the strengthening of cultural identity embedded with traditional tribal practices and Indigenous worldviews were found to be effective in mitigating risk factors such as historical trauma, poverty, and family violence (Baldwin et al., 2011; Barnes et al., 2019; R. A. Brown et al., 2021; Cwik et al., 2017; B. J. Freeman et al., 2016; Gfellner, 2016; Guilfoyle et al., 2010; Morcom, 2017; Urbaeva et al., 2017; Walters & Seymour, 2017; Woods et al., 2022). Integrated programmes based on Indigenous ways of knowing, incorporating traditional healing practices and strengthening cultural identity, family connectedness, and partnerships with community were found to be most effective in addressing issues such as substance use, mental health disorders, trauma and suicide (Allen et al., 2022; Austin et al., 1993; Baldwin et al., 2020; Brockie et al., 2022; Dickerson et al., 2016; J. Goodkind et al., 2012; Houkamau et al., 2021; Liebenberg et al., 2023; Lowe et al., 2024; Nalls et al., 2009; Payne et al., 2013; Roy et al., 2015; Shadlow et al., 2018; M. Shepherd et al., 2018; Stanley et al., 2018; Williams et al., 2018; Woods et al., 2023; Young et al., 2017). Reinforcing cultural connections showed demonstrated reductions in the negative effects of colonisation, namely, physical and relational aggression, impaired mental health and wellbeing (Blacklock et al., 2020; Flanagan et al., 2011; Rodney et al., 2005; Severinsen & Reweti, 2019; Stuart & Jose, 2014).

The review found that support, based on early interventions with a focus on strengthening cultural parenting practices that promote safe and responsive parent−child relationships, was effective in increasing the SEWB of Indigenous young people impacted by violence (Culp & McCarthick, 1997; Lee et al., 2010; Mullany et al., 2012; Twizeyemariya et al., 2017). School-based programmes were also effective in strengthening young Indigenous people’s sense of wellbeing and self-determination when incorporating bi-cultural learning and mentoring to create a culturally safe space to address trauma and grief. These programmes improved student relationships, leadership, school performance and engagement, wellbeing and cultural identity (Armstrong et al., 2022; N. Brown et al., 2023; Crooks et al., 2015, 2017; Doige, 1999; Durmush et al., 2024; Gibson et al., 2022; Louth et al., 2021; Marsiglia et al., 1998; Njeze et al., 2020; O’Brien & Bobongie-Harris, 2023; Peacock & Albert, 2000; Robinson & Tyler, 2020; K. A. Senior & Chenhall, 2012; St John & Edwards-Vandenhoek, 2022).

Resilience within an Indigenous context is grounded in culturally distinctive concepts that are inclusive of a person’s connection to community, the environment, cultural traditions, languages and a collective sense of self (Durmush et al., 2021; Fox et al., 2018; Kirmayer et al., 2003). Promoting Indigenous youth resilience by strengthening connection to community and cultural pride and engaging in traditional cultural practices enhanced wellbeing and cultural identities (Allan, 2011; Good et al., 2021; Heid et al., 2022; Lunda & Green, 2020; Stelkia et al., 2021; Whitlow et al., 2019).

Indigenous youth with a strong sense of cultural identity were shown to have positive SEWB and were less likely to engage in substance use and delinquent behaviour and had a greater capacity to cope with stressors into adulthood (Angelino et al., 2024; R. A. Brown et al., 2021; Burack et al., 2019; Garrett et al., 2014; Murrup-Stewart et al., 2021; Sahota, 2019; Wexler, 2014). Indigenous adolescents with a strong cultural identity when compared to those with a fractured construct experienced fewer symptoms of depression (Rieckmann et al., 2004) and presented with fewer behavioural and emotional problems (Fatima et al., 2022). Positive social influence, instilling cultural identity, ensuring community safeguards, assisting personal empowerment, and strengthening positive peer relationships and family connections were strong predictors of resilience and improved mental health and wellbeing among Indigenous youth (Andersson & Ledogar, 2008; Hopkins et al., 2014, 2018; Kral et al., 2014; Ponce-Garcia et al., 2019; Schanen et al., 2017; Stiffman et al., 2007; Young et al., 2017). Strong cultural connections were found to be a protective factor against suicide for both Māori and First Nations Canadian youth (Aho & Liu, 2010; Anang et al., 2019)

### Family wellbeing

Six studies demonstrated how strengthening of parenting capacity and family connections can be protective factors against the negative impacts of LV and intergenerational or historical trauma (Appendix II, Table A7). Family-based programmes based on psychosocial empowerment strategies improved SEWB and health outcomes (Gee et al., 2020; Tsey et al., 2010). Trauma interventions drawing on female family connections, with mothers and grandmothers as role models to address historical trauma, improved SEWB for young First Nations Canadian women and girls (Cooper et al., 2019). The quality of family connection, particularly parental warmth, was found to be important for Native American children’s mental health and non-engagement in substance use (Schick et al., 2022). The inclusion of Indigenous cultural content in family-based programmes was found to improve children’s SEWB, increase parental satisfaction and improve children’s challenging behaviours and decrease interparental conflict about parenting (Macvean et al., 2015) (Keown et al., 2018). Parental warmth and strong family connections were demonstrated to be protective factors against self-harm and suicidality for Aboriginal and Torres Strait Islander young people (Islam et al., 2022).

### Use of digital technology

Digital technologies emerged as intervention tools within six studies to address the impact of LV and to strengthen cultural identity and SEWB among Indigenous youth (Appendix II, Table A8). Digital technologies can facilitate healing, cultural continuity, self-determination and empowerment for young Indigenous youth to thrive (R. Walker et al., 2021). Culturally safe, online resources were used as an intervention option targeting family violence (Fiolet et al., 2020). Digital storytelling was shown to help with identity struggles, health and social issues, community connections, and bridging generational gaps when it featured the cultural teachings of Elders (S. Freeman et al., 2020; Wexler et al., 2014). Social media was shown to be a helpful mechanism for maintaining cultural knowledge and practice (Singleton et al., 2009; R. Walker et al., 2021). However, it was also deemed important to incorporate strategies to manage negative behaviours that appear on social media, such as cyber bullying, cyber racism and sexual coercion, when using this format as an intervention gateway (Rice et al., 2016).

### Trauma interventions

Five articles described culturally adapted trauma interventions designed to address adverse psychosocial outcomes due to exposure to LV (Appendix II, Table A9). Trauma-focused Cognitive Behavioural Therapy (CBT) interventions were shown to decrease anxiety and post-traumatic stress disorder symptoms in American Indian adolescents when incorporating cultural teachings, beliefs and protocols (J. R. Goodkind et al., 2010) and reduced sexualized behaviour and increased resilience for young Aboriginal and Torres Strait Islander children who had been sexually abused (Prentice et al., 2012). Prevention programmes aimed at the early detection, response, and prevention of sexual assault in Aboriginal communities were most successful when developed in collaboration between Aboriginal communities and government agencies based on proactive outreach, capacity building, holistic and integrated service, and trust (Mace et al., 2014). An exploratory study investigated a mindfulness-based prevention interventions among Native American young people (Le & Gobert, 2015). This work found positive reports of improved self-regulation, less mind wandering, and decreased suicidal thoughts associated with mindfulness-based prevention interventions (Le & Gobert, 2015). Targeted interventions for young Indigenous women found a need for cultural adaptability, early intervention, community engagement based on trust, accessibility and targeted to “at risk” groups such as Indigenous young people (Crooks et al., 2019).

## Discussion

The aim and purpose of the scoping review was to describe how young Indigenous people aged between 0 and 25 years across the CANZUS nations (Canada, Australia, New Zealand, and the United States) experience lateral violence particularly relating to the context and forms of LV; and the mechanisms of lateral empowerment pertaining to healing programmes and interventions, which address young Indigenous people’s healthy wellbeing. From the 170 peer-reviewed articles that related to LV and LE, nine topic themes emerged. Many of the themes overlapped considerably with persistent and core information about colonization, identity, young people’s voices and holistic healing. There were only two articles that used the actual term “lateral violence” for young people (Bailey, 2020; McAlister et al., 2024), and none used the term “lateral empowerment”. Although both terms are increasingly used in the adult population (Clark & Glover, 2020) and in the grey literature (Clark et al., 2024; GreyNet International, 2024), they still appear to be rarely applied to young Indigenous people when describing and talking about within-group violence. In our scoping review, it was a necessity to go beyond the terminology of “lateral violence” and seek a broader understanding of how young people conceptualize and experience LV within their communities and contexts. The themes pertaining to LV found within this scoping review reflected this aim of the study, by identifying broader terminology, forms and experiences of LV specific to Indigenous young people.

Studies under the banner of family and/or community violence indicated the cyclic nature of trauma and highlighted the negative impact of this exposure on Indigenous young people’s SEWB. Peer and gang affiliation was discussed in the literature primarily as a coping mechanism to mediate the negative impacts of family and community violence that resulted in fractured families, racism, poverty, and social disadvantage (Bell & Lim, 2005; McNulty & Bellair, 2003; Peacock & Albert, 2000). These ongoing traumatic experiences of LV within communities were the target of LE campaigns that emphasized community healing and support, many of which focused on promoting family wellbeing by enhancing parenting capacity and family connections to empower youth to break the cycle of intergenerational trauma.

The form in which young people expressed LV was found to be exhibited externally within their interpersonal relationships characterized by physical and sexual violence, dating violence and bullying. Inward expressions of LV were reflected in the form of self-harm and suicidal behaviour. Further research in this area is needed to explore both external and internal expressions of LV and what precipitates self-harm behaviours such as suicide. For example, how much of the LV behaviour is attributable to suicidal behaviour and how much might be due to other stressors or psychological distress.

Social media and technology are the dominant ways that young people interact. Its usage is a mixture of negative experiences where LV can be enacted. However, at the same time, the review found social media and technology to be a useful way to combat LV such as online digital storytelling which provides young people a platform to talk about experiences of community and family violence as well as access safe resources (S. Freeman et al., 2020; Wexler et al., 2014). These findings highlight the necessity for interventions and resources addressing LV and LE to integrate the use of digital and social media platforms as an information source for young people when disseminating this information.

The strengthening of cultural identity and resilience via connections to young people’s cultural traditions, beliefs and community was identified as integral to LE (which comprised 80 articles of the 170 results). Such connections mitigated the effects of LV and demonstrated the positive impact on their SEWB, when young people have a strong sense of their cultural identity “who they are” and are grounded in their values “what they believe” and have a strong sense of belonging to their community, land and spirituality, emphasizing the importance of incorporating these components of SEWB into intervention programmes and family and community relationships. In contrast, the study by McAlister et al. (2024) found that when Aboriginal children have a stronger sense of cultural identity, they can become vulnerable to criticism by peers. This indicates that they become more prone to LV by peers with attempts to diminish their confidence and sense of identity. Hence, findings such as these need to be considered in interventions that attempt to bolster cultural identity and resilience for LV and LE.

Furthermore, a proportion of the literature focused on coping behaviours with many preventative and early intervention processes, demonstrating that there is an incredible capacity for healing, learning, and adapting that goes beyond coping. Many of the studies described a diversity of efforts to laterally empower young people to address LV within their communities and families and promote resilience through interventions and programmes focused on the integration of cultural practices. This was used to facilitate strong community connections, heal family relationships, create positive peer relationships, and strengthen cultural identity as mechanisms to counteract the negative impacts of LV (Caputo & Kelly, 2015; Gazis et al., 2010; Hopkins et al., 2014, 2018; Scott & Dearing, 2012; Young et al., 2017). While the research demonstrates that young Indigenous people across the CANZUS nations experience many types of violence in their homes, communities and peer relationships, it also highlights their strength, agency, and the importance of culturally grounded, community-led responses that prioritize self-determination, connection to culture, and holistic wellbeing. These approaches not only support recovery but also foster environments where young people can thrive despite the ongoing challenges posed by lateral violence and other negative influences. The literature was hopeful and highlighted a great capacity for overcoming the effects of violence, through strength-based empowering practices that incorporate not just the individual but communities, families and peers.

Effective interventions indicated the incorporation of Indigenous perspectives and leadership, acknowledging the ongoing impacts of colonization on families and communities. There is a need for awareness, education, support, relationship building and connectivity to various SEWB domains as well as enabling community and young people’s voices in the development of purposeful and tailored interventions, including community-initiated and informed solutions (Meyer & Stambe, 2022; Noble-Carr et al., 2019; Taylor et al., 2004). Through empowerment, such interventions are better able to assist in breaking the cycle of intergenerational violence to address the “wounding pattern of historical oppression” (Jaber et al., 2023, p. 12).

### Strengths and limitations

A notable asset is that the scoping review took a strength-based approach and identified a breadth of research aimed at rebuilding communities and strengthening families impacted by ongoing colonialism, racism, and oppression.

Through the application of broad search terms that were relational to LV and LE, alternative and conceptually linked terminology was captured, rather than relying on specific terms such as “lateral violence”. In fact, LV was rarely used in the resulting publications about Indigenous young people. However, through this process, it is possible that other relational terms may have been unintentionally left out or missed in the search. For example, it would not be feasible to include all region-specific and specific cultural terms that might be used to describe the phenomena of LV and LE in a variety of young people’s contexts. The intention of the review was to privilege the voices and experiences of young Indigenous people across the CANZUS nations, regardless of how they may refer to LV or LE. Another issue within the literature was that the indigeneity of some of the participants was not reported. For example, papers describing experiences of dating violence against Indigenous girls and young women may not have specified the cultural backgrounds of the perpetrators of violence. Despite this, our approach was to be overinclusive to ensure that in the event where cultural background was specified that these studies were included to ensure the review captured a complete, holistic picture of the experiences and impacts of violence within communities of Indigenous young people.

Consequently, the review included many peer-reviewed articles beyond that of a standard scoping review. This scoping review was done in tandem with a grey literature scoping review which meant that the number of overall articles for this topic exceeded the 170 articles found in this review. We divided the scoping reviews into two and the grey literature material has been reported elsewhere (Clark et al., 2024). The large number meant that a thorough and necessary map of the literature pertaining to LV and LE was achievable, however this was at the expense of the depth of the findings.

Although the study omitted the use of the CREATE Aboriginal and Torres Strait Islander Quality Appraisal Tool (Harfield et al., 2020) this scoping review was still able to highlight Indigeneity and the privileging of Indigenous voices in the literature. Furthermore, there was demonstration of a plethora of strength-based findings about programmes and interventions for young Indigenous people that foster their strength and capacity building to thrive and overcome the negative impacts of LV

### Implications and directions for future research

This scoping review identified several areas for further research related to LV and LE among young Indigenous peoples. Whilst there were many articles about family and domestic violence and the experiences and impacts on young Indigenous people in our scoping review, this review found only limited research about other forms of LV such as suicide exposure, dating violence, and deviant peer affiliation. A broader understanding of the variety of ways in which LV is manifested and experienced among young Indigenous people is necessary to design effective, community-informed strategies to address the various forms of LV. Further research that explores young peoples’ attitudes, experiences, directions, opinions about healing and recovery would likewise be beneficial.

It is crucial that research related to Indigenous peoples reflects the strength of Indigenous cultural values and concepts such as connectedness, collectivism, and diversity, and empowers the voices and agencies of young Indigenous people. All research with Indigenous peoples should be community sanctioned and led, utilizing the wisdom of Elders, spiritual leaders, community members, family, and most importantly, young people themselves (Payne et al., 2013).

## Conclusion

Young Indigenous people within the CANZUS nations are experiencing lateral violence in many forms and is relational to other phenomena such as racism, oppression, trauma, psychological distress and poverty. There is a growing corpus of strength-based research that focuses on resilience and healing within Indigenous communities. This scoping review has highlighted a variety of empowering strategies that empower and privilege the voices of young people to strengthen their wellbeing, cultural identity, a sense of belonging and connectedness to family, community, and country. It will be important to extend this review with vital research, yarning and learning from young people via co-design and co-development of healing and wellbeing programmes for young Indigenous people.

## Data Availability

The authors confirm that the data supporting the findings are found within the article. Any additional data can be made available upon reasonable request to the first author.

## Appendix I. Data extraction instrument

**Table:**

Heading	Description
Study Information	
Title of article	What is the title of the article or source?
Year of publication	<2000, 2000–2010, 2011–2017, 2018–2022
Indigenous population	Which Indigenous population did the study refer to?
Country of origin	What is the country of origin where the study was conducted
Source type	Type of research article: original research, review, thesis, etc.
Citation details	Author, A.A., & Author, B.B., (Year). Title of the article. Name of the journal, volume(issue), #-#. https://doi.org/xxx
What is the article about	Lateral Violence, Lateral Empowerment, or both.
Concept	What type of LV/LE was the study about?
Methodology	What was the type of study used in this article (pilot study, interviews, RCT, literature review etc.?)
Aims/purpose/objective	What is the identified objective, aim, or purpose of the study?
Context – study setting	Where was the study conducted?
Participant characteristics (*n*=)	Who were the participants of the study? How many participants were there in the study? What were the participant characteristics
Outcome of the study	What was the outcome of the study? How did they measure the outcome?
Key findings and outcomes	What are the key findings and outcomes of the study?
Limitations or challenges	What limitations did the author report? Were there any other major limitations or challenges?
Gaps and future direction	What are the gaps identified by the researchers? What are the recommendations for future research?
Conclusion	What are the overall conclusions of the article?

## Appendix II. Theme Tables

### Lateral Violence Tables

**Table A1.** Familial and/or community violence.

Country	Year published	Sample size range	Research design	Study setting	Population Characteristics	% participants that are Indigenous	First Author
AUS n = 8	1 2000–2010 6 2011–2021 1 2022–2024	n = 17–49,953	2 Quant 6 Qual 0 Review	6 Community 2 Other	Ages 0–25	2 52–53% Indigenous 4 100% Indigenous 2 Not disclosed	Askew et al., (2013); Basu & Isaacs, ((2019); Cripps et al., (2009); Frost, (2014); Meyer & Stambe, (2022); Noble-Carr et al., (2019); Orr et al., (2020); Soldatic et al., (2023)
CAN n = 2	1 2000–2010 1 2011–2017	n = 23–12,366	1 Quant 1 Qual	1 Community 1 School	Ages 7–19	2 100% Indigenous	Coleman et al., (2001); Kaspar, (2013)
NZ n = 8	3 2000–2010 5 2011–2021	n = 5–2,887	4 Quant 3 Qual 1 Review	4 Community 4 Other	Ages 10–21	4 11–66% Indigenous 3 100% Indigenous 1 Not disclosed	Fanslow et al., (2021); Ioane & Lambie, (2016); Ioane et al., (2014); Lim et al., (2012); Marie et al., (2009); Ryan & Wilson, (2010); Short et al., (2019); Wilson et al., (2004)
USA n = 11	6 2000–2010 5 2011–2021	n = 4–18,868	5 Quant 4 Qual 2 Review	6 Community 3 School 1 Service 1 Other	Ages 0–21	5 9–97% Indigenous 6 100% Indigenous	Bearinger et al., (2005); Dickerson & Johnson, (2012); Hautala & Sittner, (2021); Linton & Kim, (2014); Matthay et al., (2019); McKinney, (2003); McNulty & Bellair, (2003)Nalls et al., (2009); Probst et al., (2008); Sarche et al., (2009); Willmon-Haque & Bigfoot, (2008)

**Table A2.** Interpersonal violence victimization (IVV).

Country	No of studies by year published	Sample size range	Research design	Study setting	Population Characteristics	No of studies and % participants that are Indigenous	First Author
AUS n = 5	4 2000–2010 1 2011–2017	n = 88–755,785	4 Quant 1 Qual 1 Mixed	3 Community 2 Other	Ages 0–18	3 10–65% Indigenous 2 100% Indigenous	Berry et al., (2009); Meuleners et al., (2009), (2010); K. Senior et al., (2017); Shore & Spicer, (2004)
CAN n = 1	1 2011–2017	n = 948	1 Qual	1 Other	Ages 12–25	1 12% Indigenous	Du Mont et al., (2017)
NZ n = 3	3 2018–2023	n = 6–200	1 Quant 2 Qual	33 Community 1 Other	Ages 10–19	3 100% Indigenous	Dhunna et al., (2021); Grey et al., (2023); Short et al., (2019)
USA n = 5	2 2000–2010 3 2018–2021	n = 94–88,219	2 Quant 3 Qual	3 School 2 Community	Ages 9–25	2 5–15% Indigenous 2 100% Indigenous 1 Not disclosed	Andreescu & Overstreet, (2020); Edwards et al., (2021); Fix et al., (2021); Oetzel & Duran, (2004); Rodney et al., (2005)

**Table A3.** Peer and gang violence.

Country	No of studies by year published	Sample size range	Research design	Study setting	Population Characteristics	No of studies and % participants that are Indigenous	First Author
AUS n = 1	1 2000–2010	n = 274	1 Qual	1 School	Ages 12–16	1 32% Indigenous	Gazis et al., (2010)
CAN n = 2	2 2011–2021	n = 12,366	1 Qual	1 School	Ages 6–18	1 100% Indigenous	Bailey, (2020); Kaspar, (2013)
NZ n = 1	1 2011–2017	n = 1,265	1 Mixed	1 Other	Ages 0–25	1 10% Indigenous	Gillies et al., (2017)
USA n = 5	4 2000–2010 1 2011–2017	n = 36–13,460	1 Quant 4 Qual	2 Community 3 School	Ages 11–20	1 Not disclosed 4 100% Indigenous	Bell & Lim, (2005); Donnermeyer et al., (2000); McNulty & Bellair, (2003); Peacock & Albert, (2000); Scott & Dearing, (2012)

**Table A4.** Self-harm behaviours.

Country	No of studies by year published	Sample size range	Research design	Study setting	Population Characteristics	No of studies and % participants that are Indigenous	First Author
AUS n = 1	1 2020–2024	n = 117	1 Quant	1 Coronial data	Ages 10–24	1 48% Indigenous	Hill et al., (2020)
CAN n = 1	1 2011–2017	n/a	1 Qual	1 Community	n/a	1 100% Indigenous	Kral, (2012)
NZ n = 1	1 2000–2010	n/a	1 Qual	1 Community	n/a	100% Indigenous	Aho & Liu, (2010)
USA n = 3	2 2000–2010 1 2011–2017	n = 314–881	1 Quant 2 Qual	2 School 1 Community	Ages 13–20	1 22% Indigenous 2 100% Indigenous	Else et al., (2009); Freedenthal & Stiffman, (2004); Wexler et al., (2012)
CANZUS n = 1	1 2011–2017	n/a	1 Qual	1 Review	n/a	100% Indigenous	Hatcher et al., (2017)

### Lateral Empowerment Tables

**Table A5.** Community informed treatment approach.

Country	No of studies by year published	Sample size range	Research design	Study setting	Population Characteristics	No of studies and % participants that are Indigenous	First Author
AUS n = 2	1 2000–2010 1 2011–2021	n = 6	2 Qual	1 Community 1 Service	Ages 35–55 (parents of young people)	2 100% Indigenous	Gee et al., (2020); Taylor et al., (2004)
CAN n = 3	1 2000–2010 2 2011–2021	n = 461	2 Qual 1 Qual	2 Community 1 School	Ages 0–25	3 100% Indigenous	DeRiviere, (2018); Kral et al., (2014); Zahradnik et al., (2010)
NZ n = 1	1 2000–2010	n/a	1 Review	1 Community		1 100% Indigenous	Taonui, (2010)
USA n = 7	1 2000–2010 5 2011–2021 1 2021– 2024	n = 67–432	4 Quant 3 Qual	4 Community 2 school 1 Mental Health Service	Ages 6–24	1 11% Indigenous 6 100% Indigenous	Dickerson & Johnson, (2011); Komro et al., (2023); McWhirter, (2010); O’Keefe et al., (2019); Rothman et al., (2022); Trout et al., (2018); Wexler et al., (2012)

**Table A6.** Cultural identity and resilience.

Country	No of studies by year published	Sample size range	Research design	Study setting	Population Characteristics	No of studies and % participants that are Indigenous	First Author
AUS n = 19	2 2000–2010 10 2011–2021 7 2022–2024	n = 3–1671	16 Qual 1 Mixed 1 Review 1 Other	6 Community 7 School 1 Family 5 Other	Ages 0–23	16 100% Indigenous 3 Not disclosed	Armstrong et al., (2022); N. Brown et al., (2023); Durmush et al., (2021), (2024); Fatima et al., (2022); Gibson et al., (2022); Guilfoyle et al., (2010); Hopkins et al., (2014), (2018); Lee et al., (2010); Louth et al., (2021); McAlister et al., (2024); Murrup-Stewart et al., (2021); O’Brien & Bobongie-Harris, (2023); Robinson & Tyler, (2020); K. A. Senior & Chenhall, (2012); St John & Edwards-Vandenhoek, (2022); Twizeyemariya et al., (2017); Young et al., (2017)
CAN *N* = 18	1 < 2000 1 2000–2010 10 2011–2020 5 2021–2024	n = 14–126	11 Qual 4 Reviews 1 Evaluation 1 Case Report	8 Community 8 School 1 Other	Ages 7–24	3 50–95% Indigenous 14 100% Indigenous	Anang et al., (2019); Andersson & Ledogar, (2008); Blacklock et al., (2020); Burack et al., (2019); Crooks et al., (2015), (2017); Doige, (1999); Flanagan et al., (2011); Good et al., (2021); Heid et al., (2022); Kirmayer et al., (2003); Kral et al., (2014); Liebenberg et al., (2023); Morcom, (2017); Nightingale & Richmond, (2022); Njeze et al., (2020); Stelkia et al., (2021); Whitlow et al., (2019)
NZ n = 9	1 2000–2010 8 2011–2021	n = 6–3803	1 Quant 6 Qual 2 Mixed methods	3 Community 5 School 1 Other	Ages 9–18	9 100% Indigenous	Aho & Liu, (2010); Barnes et al., (2019); Fox et al., (2018); Houkamau et al., (2021); Severinsen & Reweti, (2019); M. Shepherd et al., (2018); Stuart & Jose, (2014); Walters & Seymour, (2017); Williams et al., (2018)
USA n = 33	3 < 2000 4 2000–2010 22 2011–2021 5 2022–2024	n = 6–2,548	3 Quant 23 Qual 1 Mixed 4 Review 1 Thesis 2 Case Report	20 Community 9 School 5 Other	Ages 2–20	4 15–97% Indigenous 27 100% Indigenous 3 Not disclosed	Allan, (2011); Allen et al., (2022); Angelino et al., (2024); Austin et al., (1993); Baldwin et al., (2020); Brockie et al., (2022); R. A. Brown et al., (2021); Culp & McCarthick, (1997); Cwik et al., (2017); Dickerson et al., (2016); B. J. Freeman et al., (2016); Garrett et al., (2014); Gfellner, (2016); J. Goodkind et al., (2012); Lowe et al., (2024); Lunda & Green, (2020); Marsiglia et al., (1998); Mullany et al., (2012)Nalls et al., (2009); Payne et al., (2013); Peacock & Albert, (2000); Ponce-Garcia et al., (2019); Rieckmann et al., (2004); Rodney et al., (2005); Sahota, (2019); Schanen et al., (2017); Shadlow et al., (2018); Stanley et al., (2018); Stiffman et al., (2007); Urbaeva et al., (2017); Wexler, (2014); Woods et al., (2022), (2023)
CANZUS n = 1	1 2011–2017	n = 16 articles	1 Review	1 Community	Ages 12–29	1 100% Indigenous	Roy et al., (2015)

**Table A7.** Family wellbeing.

Country	No of studies by year published	Sample size range	Research design	Study setting	Population Characteristics	No of studies and % participants that are Indigenous	First Author
AUS n = 3	1 2000–2010 1 2011–2021 1 2022–2024	n = 6–218	3 Qual	1 School 1 Service 1 Community	Ages 5–18 (children) Ages 35–55 (parents)	3 100% Indigenous	Gee et al., (2020); Islam et al., (2022); Tsey et al., (2010)
CAN n = 1	1 2018–2021	n = 60	1 Qual	1 Community	Ages 8–12	1 100% Indigenous	Cooper et al., (2019)
NZ n = 1	1 2018–2021	n = 70	1 Quant	1 School	Parents and young children	1 100% Indigenous	Keown et al., (2018)
CANZUS n = 2	2 2011–2022	n/a	2 Qual	2 School	Parents	2 100% Indigenous	Macvean et al., (2015); Schick et al., (2022)

**Table A8.** Use of digital technology.

Country	No of studies by year published	Sample size range	Research design	Study setting	Population Characteristics	No of studies and % participants that are Indigenous	First Author
AUS n = 4	1 2000–2010 3 2011–2021	n = 23 (1 study)	2 Qual 2 Review	3 Community 1 School 1 Other 1 Service	Ages 7–25	4 100% Indigenous	(Fiolet et al., (2020); Rice et al., (2016); Singleton et al., (2009); R. Walker et al., (2021)
CAN n = 1	1 2018–2021	n = 116	1 Qual	1 Community	High School Students	1 100% Indigenous	(S. Freeman et al., (2020)
USA n = 1	1 2011–2017	n = 271	1 Qual	1 School	Ages 9–19	1 100% Indigenous	(Wexler et al., (2014)

**Table A9.** Trauma intervention.

Country	No of studies by year published	Sample size range	Research design	Study setting	Population Characteristics	No of studies and % participants that are Indigenous	First Author
AUS n = 2	2 2011–2017	n = 64–65	1 Qual 1 Evaluation	1 Community 1 School	Ages 3–6 (1 study)	1 9% Indigenous 1 Not disclosed	(Mace et al., (2014); Prentice et al., (2012)
CAN n = 1	1 2018–2021	n/a	1 Review	1 Community	Ages 12–24	1 Not disclosed	(Crooks et al., (2019)
USA n = 2	2 2000–2015	n = 23	1 Mixed 1 Qual	2 School	6–8^th^ grade Ages 15–20	2 100% Indigenous	(J. R. Goodkind et al., (2010); Le & Gobert, (2015)
